# Simplified 3D protocol capable of generating early cortical neuroepithelium

**DOI:** 10.1242/bio.021725

**Published:** 2017-02-06

**Authors:** Dwayne B. Holmes, Vivi M. Heine

**Affiliations:** 1 Department of Pediatrics/Child Neurology, Amsterdam Neuroscience, VU University Medical Center, Amsterdam 1081 HV, The Netherlands; 2 Department of Complex Trait Genetics, Center for Neurogenomics and Cognitive Research, Amsterdam Neuroscience, VU University, Amsterdam 1081 HV, The Netherlands

**Keywords:** 3D, Organoid, Pluripotent stem cell, Cerebellum, Cortex

## Abstract

Here, we report a 3D cerebellar differentiation protocol with quick startup method, defined medium and no special materials or handling requirements. Three fibroblast growth factors (FGF2, 4 and 8) were used for cerebellar patterning and smoothened agonist (SAG) for granule cell development. After 35 days, differentiation products exhibited similar structures and neuronal markers reported in prior ‘organoid’ and ‘spheroid’ protocols. This included cells positive for KIRREL2 (a marker of early cerebellar neuroepithelium) and ZIC1 (a marker for granule cells). Follow-up tests indicated that addition of FGFs, if helpful, was not required to generate observed structures and cell types. This suggests that intrinsic production of patterning factors by aggregates themselves may be adequate for region-specific 3D modeling. This protocol may be used as a quick, easy and cost-efficient method for 3D culture, whether to research development of the early cerebellar neuroepithelium, a base to generate mature cortical structures, or to optimize minimal-factor protocols for other brain regions.

## Abstract (precis)

**Summary:** We present a simplified method to generate early neuroepithelium of the cerebellar cortex in a 3D culture using defined medium and minimal addition of growth factors.

## INTRODUCTION

Protocols for modeling brain development, neural disorders, and brain evolution *in vitro* using pluripotent stem cells (PSCs) are beginning to shift away from adherent, two-dimensional (2D) cultures toward free-floating, three-dimensional (3D) ‘organoid’ cultures (Karus et al., 2014; Kelava and Lancaster, 2016). The main reason for this is the potential 3D environments offer for reproducing architectures and mechanisms not possible in 2D monolayers. 3D protocols have already been used to model forebrain cortical structures (Kadoshima et al., 2013; Lancaster et al., 2013; Otani et al., 2016; Pasca et al., 2015), as well as specific brain regions such as the hypothalamus and cerebellum (Muguruma et al., 2015; Qian et al., 2016). However, 3D cultures are often more complex and costly than 2D culture systems.

Developmental studies have shown that generation of mid-hindbrain structures involve coordinated activity of different signaling molecules (reviewed by Tam et al., 2017). WNT, BMP and fibroblast growth factors (FGFs) in particular set pro-cerebellar patterning of the mid-hindbrain region (Joyner, 1996) and guide formation of the isthmic organizer (Joyner et al., 2000). Addition of FGF8 and retinoic acid (RA) was reported to be sufficient to induce gene expression profiles suggestive of the cerebellar anlagen in a 2D *in vitro* differentiation protocol (Erceg et al., 2012, 2010), while that and similar protocols used FGF2 and FGF4, in combination with various WNT and BMP proteins, to complete differentiation (Salero and Hatten, 2007; Su et al., 2006). Sonic hedgehog (SHH), produced by Purkinje cells *in vivo*, is also known to be important for granule cell precursor (GCP) growth in the developing cerebellar cortex (Dahmane and Ruiz i Altaba, 1999). As such, it was used in the final maturation step by Erceg et al. (2010, 2012). In contrast to these 2D studies, a recent 3D cerebellar differentiation protocol used fewer factors for patterning, specifically FGF2, FGF19 and SDF1 (Muguruma et al., 2015).

This report describes a simple 3D protocol for modeling development of the early cerebellar cortex with human embryonic stem cells (ESCs) and induced pluripotent stem cells (iPSCs). Using defined medium in anti-adhesive-coated six-well plates (6WPs), we tested the ability of three FGFs (2, 4 and 8) and smoothened agonist (SAG) (Chen et al., 2002a,b) – a chemical agonist of the SHH pathway – to generate early cerebellar structures and granule cells in 3D culture. Our protocol successfully produced 3D cell aggregates from PSCs that exhibit markers and structures similar to those reported for other 3D protocols. Intriguingly, our results indicate that addition of FGFs was not required, suggesting that intrinsic production of patterning factors may be sufficient for 3D *in vitro* models.

## RESULTS AND DISCUSSION

### 3D products exhibit typical organoid structures and neuronal markers of neuronal induction

**Fig. 1.**
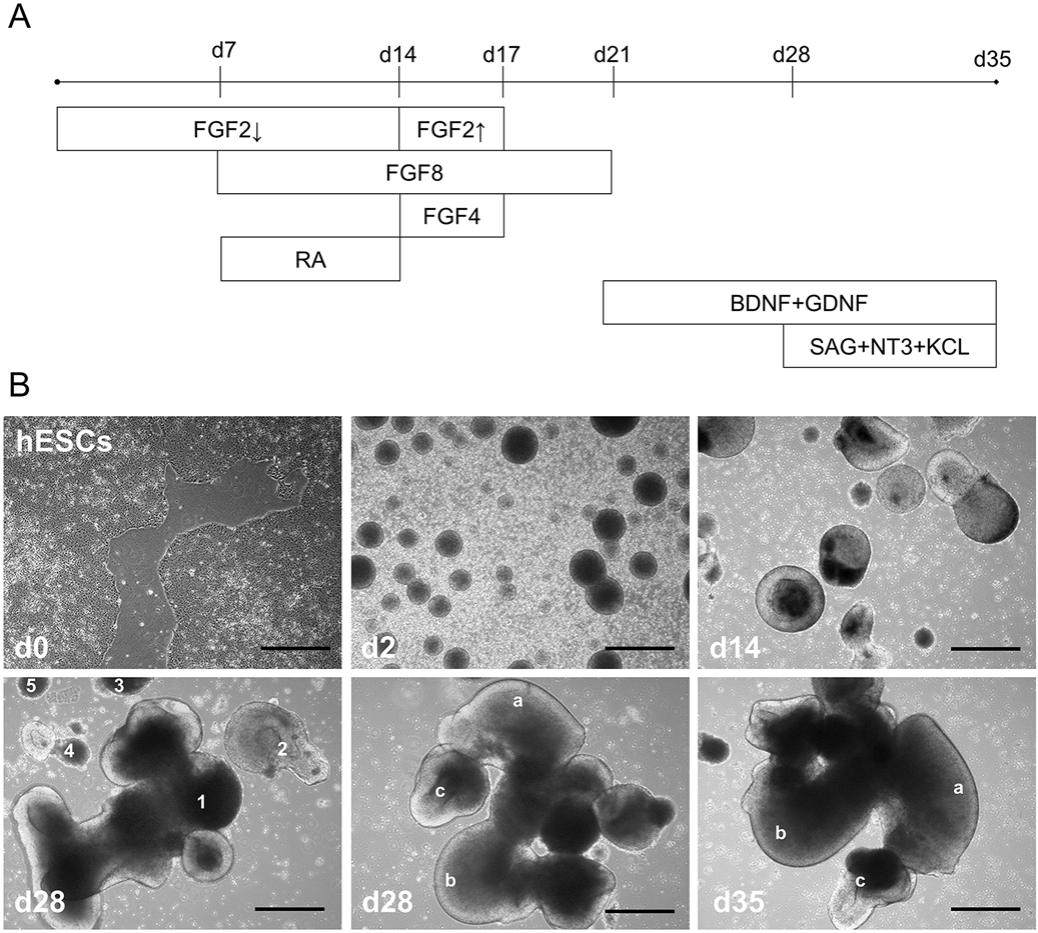
**Pro-cerebellar signaling molecules induce complex 3D organoid structures.** (A) Timeline of protocol indicating what day specific factors were added to culture medium. For FGF2 arrows refer to lower (4 ng ml^−1^, down arrow) and higher (20 ng ml^−1^, up arrow) concentrations. (B) Bright-field images show typical examples of cultures at: d0 (as ESC colonies), d2 (EBs), d14 (after induction with RA+FGF8), d28 [after patterning/before maturation, with aggregates (indicated by numbers 1-5) varying in size and complexity], d28 (a large aggregate with complex features identified by letters a-c), d35 (same aggregate from d28 with same features indicated by letters a-c allowing comparison after 7 day maturation step with SAG). Scale bars: 100 μm.

To test a simplified 3D protocol for modeling development of the early cerebellar cortex, human PSCs were differentiated in anti-adhesive plates using three growth factors (FGF2, 4 and 8) to induce proper patterning, and SAG, neurotrophic factor 3 (NT3), and brain-derived neurotrophic factor (BDNF) to assist growth and maturation of granule cells (GCs) (Borghesani et al., 2002; Chen et al., 2002a,b; Dahmane and Ruiz i Altaba, 1999) (Fig. 1A). Visual observation showed that individual aggregates tended to increase in size and complexity over the course of differentiation (Fig. 1B). However, some aggregates appeared to merge into larger bodies while others broke into smaller ones (during medium change or plate transfer), increasing variation in the number, size, and complexity of aggregates throughout cell culture (Fig. S1). As such, the protocol described here faces similar challenges due to product variability as those already reported in the field of 3D neuronal cell culture (Kelava and Lancaster, 2016).

Despite variability in aggregate size, our protocol was robust with starting populations at day (d)0-3>500 EBs, and allowed for qualitative analysis for the presence of cell types and structures. By the end of the differentiation (d35) all aggregate products per cell line were fixed, cryopreserved and embedded together. As such, every experiment included a number of aggregates that were analyzed. By analyzing all aggregates together we reduced the chance that product number or size was a confounding variable. Of note, given that not all aggregates settled at the same level or orientation, they were not cut with the same degree of representation. And, given the nature of the shape and size of aggregates, a cross-section of one aggregate might have appeared as more than one aggregate-section on a slide. We successfully repeated the experiments on human ESCs (five independent differentiations) and on three control iPSC lines (4, 3 and 1 independent differentiations, respectively).

**Fig. 2.**
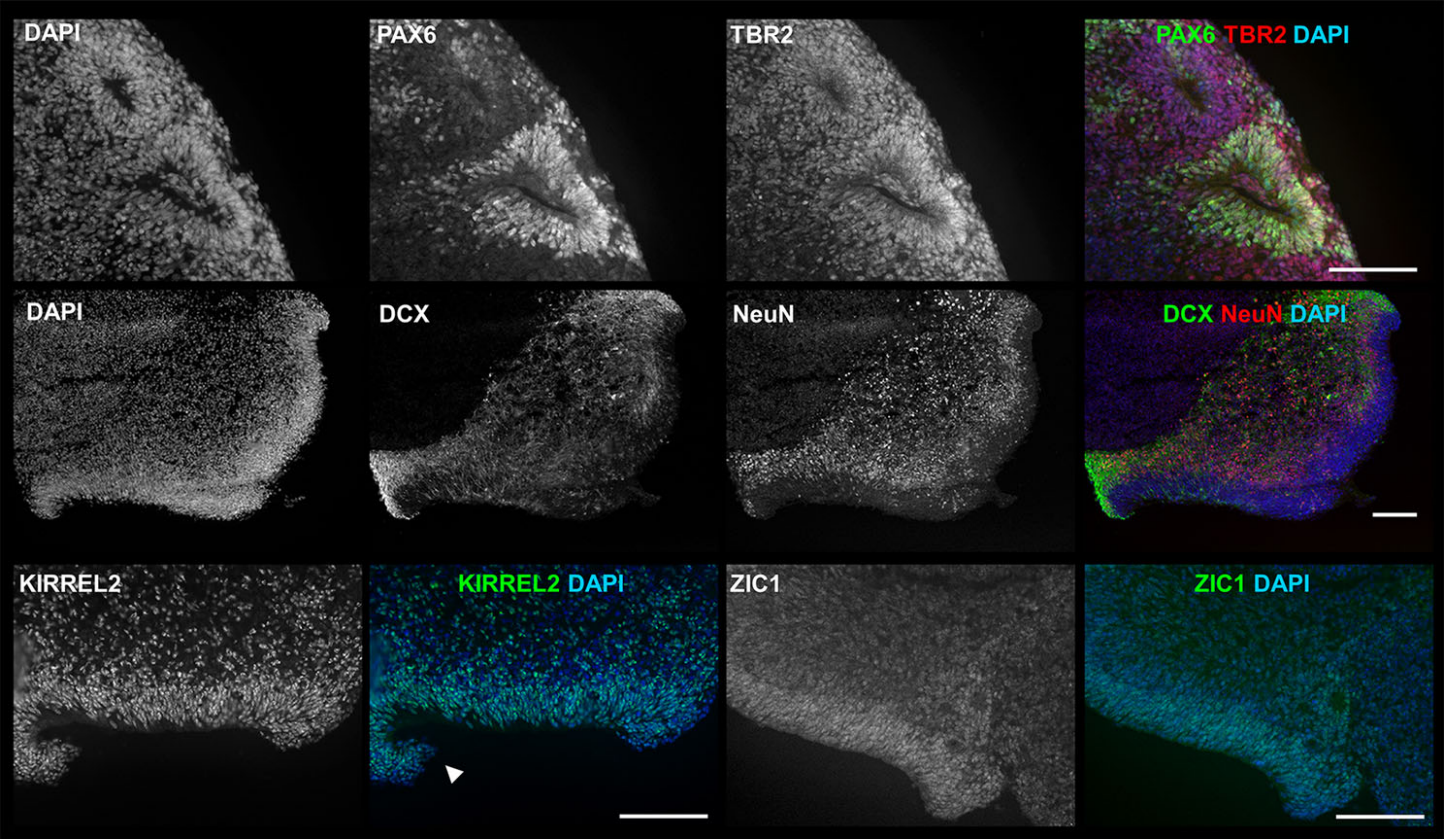
**3D products exhibit markers of early neural, neuronal and cerebellar cell types as well as purported cortical structures.** ICC analysis showed that 3D products (d35) are all positive for the early neural markers PAX6 (green) and TBR2 (red) near lumen of neural rosette-like structures (first row), the neuronal markers DCX (green) and NeuN (red) located towards interior of aggregate from outer edge ventral zone (VZ)-like structure (second row), cerebellar neuroepithelium marker KIRREL2 (third row, left) and granule cell marker ZIC1 (third row, right). Rhombic lip (RL)-like structure is indicated by arrowhead. Experiment was conducted on hESC line H01 (*n*=5), and iPSC lines hvs88 (*n*=4), hvs60 (*n*=3) and hvs51 (*n*=1). Sample images shown are from iPSC line hvs60. Scale bars: 100 μm.

To detect the presence of early neural and neuronal cell types, samples were collected and characterized by immunocytochemistry (ICC) at day 35 (Fig. 2). Cells positive for early neural markers PAX6 and/or TBR2 were observed in all aggregates. The PAX6- and TBR2-positive cells are surrounding (and seemingly spreading out from) the lumen of structures resembling neural rosettes (NRs) (Fig. 2, first row) or within and spreading out from thick layers of cells lining aggregate exteriors that have been described in literature as representing apical surfaces of ventral zones (VZs) (Kadoshima et al., 2013; Muguruma et al., 2015) (Fig. S2). Cells positive for early neuronal markers Doublecortin (DCX) or NeuN were observed in all aggregates and were located just outside the same purported NRs and VZs (Fig. 2, second row). Their location and relative maturity (DCX+ cells being less mature than NeuN+ cells) suggest neurons maturing as cells migrate from NRs and VZs to spread across the surface or toward interiors of aggregates. Although marker expression and size of structures were not uniform across all products, all markers and structures described above were observed in products for all PSC lines.

As PAX6 and TBR2 are associated *in vivo* with cells of the VZ and sub-ventral zone (SVZ), respectively (Englund et al., 2005), our results support prior identification of noted *in vitro* structures as NRs or VZ/SVZs, with apical faces of VZs turned towards the outside of aggregates (Kadoshima et al., 2013; Kelava and Lancaster, 2016; Muguruma et al., 2015). Also, the presence of curled back tips of some VZ-like regions were suggestive of the ‘asymmetric rounding morphogenesis’ of cortical neuroepithelium (NE) reported by (Kadoshima et al., 2013).

Determining whether the differentiation products described above count as ‘organoids’ is less straightforward. Using Kelava and Lancaster's cross-study visual comparison of aggregate complexity and estimated VZ/SVZ size (Fig. 3 in Kelava and Lancaster, 2016), our protocol generated aggregates (by day 35) that fit within a range from large ‘neural rosettes’ to ‘cerebral organoids’. Depending on criteria, most resemble the ‘spheroids’ generated by another simplified 3D protocol that mimicked development of the cerebral cortex (Pasca et al., 2015). It is not known if extended culture would result in larger, more complex products.

### 3D products adopt potential cerebellar identities

To check for the presence of cerebellar structures and cerebellar GCs, cultures were stained for KIRREL2 (a marker for cerebellar NE) and ZIC1 (a marker for post-mitotic GCs). KIRREL2+ cells were present in all aggregates and associated with VZ-like structures (Fig. 2), often resembling the cerebellar NE with associated rhombic lip-like feature (Fig. 2, third row, left) as reported earlier (Fig. S5P in Muguruma et al., 2015). ZIC1+ cells were found within or around NRs and VZs (Fig. 2 third row, right) in all aggregates. Since ZIC1 also labels early neural stem cells, their identification as GCs is not definitive. ZIC1-postive signal further involved low to intense staining, which could indicate different developmental stages. However, the expression of cerebellar associated markers within or around early cerebellar-associated features suggests development of cerebellar-specific structures.

### Fibroblast growth factors not required for organoid formation

**Fig. 3.**
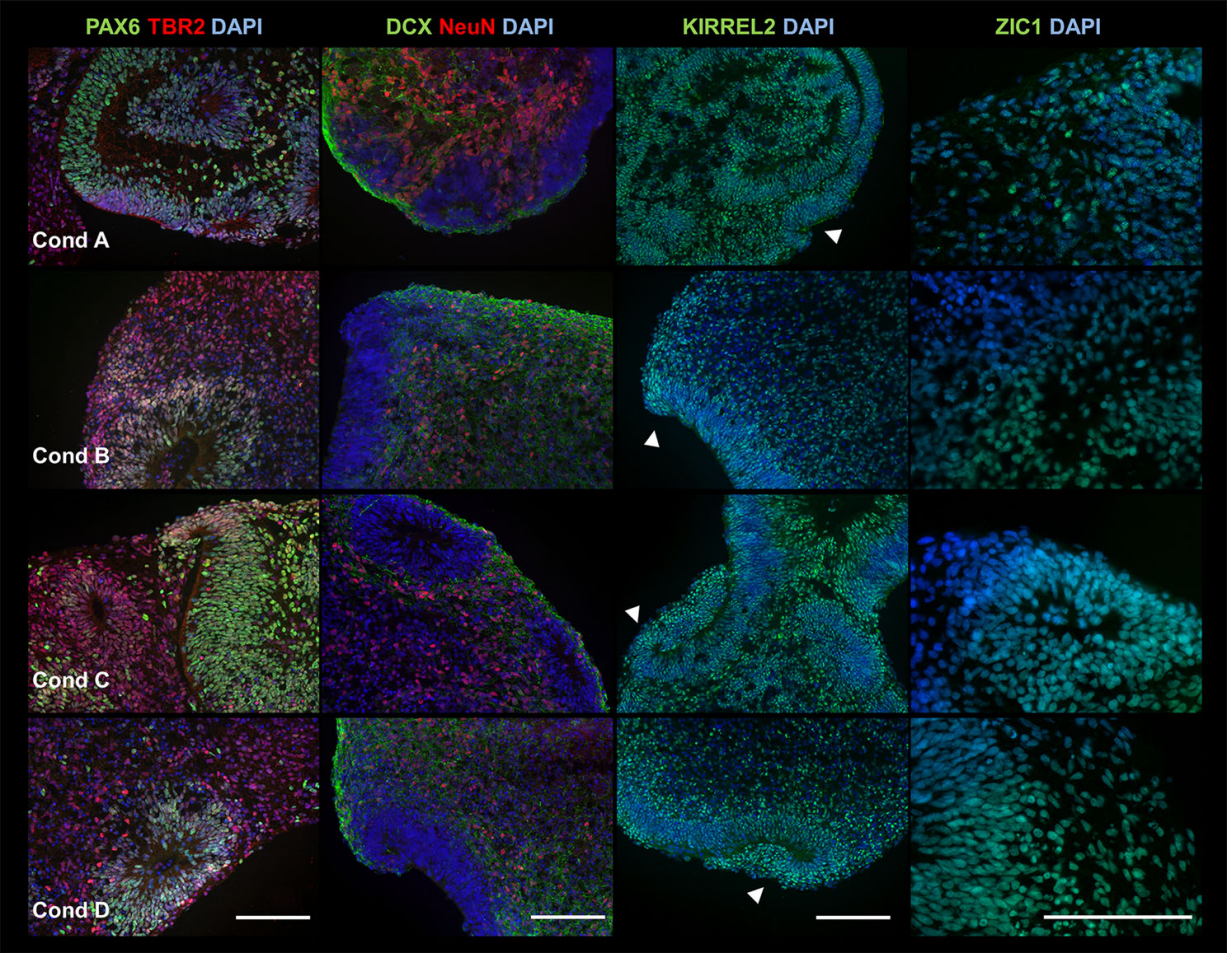
**FGFs are not required to generate early cortical neuroepithelium.** ICC analysis showed that 3D products from medium conditions A-D (rows 1-4, respectively) are all positive for early neural markers PAX6 (green) and TBR2 (red) (first column), neuronal markers DCX (green) and NeuN (red) (second column), and cerebellar neuroepithelium marker KIRREL2 (third column), and granule cell marker ZIC1 (fourth row). Rhombic lip (RL)-like structures are indicated by arrowheads. Experiment was conducted using hESC line H01 and performed three times (*n*=3) for each condition (A-D). We analyzed 2-3 serial sections per condition, which in total contained the following number of cross sections from a collection of <10 aggregates (one aggregate can consist of several merged structures), condition A (PAX6, 15; TBR2, 15; DCX, 13; NeuN, 13; KIRREL2, 12; ZIC1, 12), condition B (PAX6, 30; TBR2, 30; DCX, 27; NeuN, 27; KIRREL2, 28; ZIC1, 26), condition C (PAX6, 15; TBR2, 15; DCX, 11; NeuN, 11; KIRREL2, 10; ZIC1, 12) and condition D (PAX6, 21; TBR2, 21; DCX, 23; NeuN, 23; KIRREL2, 21; ZIC1, 20). Sample images shown are from hESC line H01. Scale bar: 100 μm.

Muguruma et al. (2015) presented a 3D cerebellar protocol and suggested that FGF2 may be sufficient to induce production of FGF8. If true, addition of FGF8 and FGF2 together would be redundant. To test the importance of different FGFs used in our protocol (FGF2, 4 and 8), ESCs were differentiated using four separate medium conditions in parallel (the rest of the protocol remaining the same). Condition A contained no FGFs, Condition B contained FGF 4 and 8, Condition C contained only FGF2, and Condition D contained FGF2, 4 and 8 (identical to the original protocol; Fig. 1A). Products were collected at day 35, with cell identities and structures characterized by ICC (Fig. 3).

Surprisingly, despite wide variation in products between experiments, similar cell types and structures were observed across all conditions, arguing for a high reproducibility. This suggests that addition of FGFs were not required to generate general neuroepithelial identities, and that (given sufficient nutrient support) developing cell aggregates may be ‘competent’ to produce their own factors for growth and patterning. However, the contribution of RA during initial neural induction, and SAG during final maturation, to final products was not tested.

Our ability to reproduce these markers using medium without FGFs (condition A) raises questions of why our results contradict in part those reported by Muguruma et al. (2015) where lack of FGF2 significantly decreased the presence of certain markers. Although we cannot explain this disagreement definitively, there are a number of differences between our materials and protocols. We feel the following are the most notable ones. To start, Muguruma et al. (2015) used an aggregation technique that kept aggregates isolated from each other (individual wells of a 96-well plate) for the first 21 days, while ours was open and allowed them to interact chemically/physically. Then there are the differences in medium additives, where we used retinoic acid to push neural differentiation and they used SB431542 (a SMAD inhibitor). And finally there were differences in the base medium itself, where we used a Neurobasal/N2+B27 medium throughout and they only switched to a Neurobasal/N2 medium on day 21 (and removed insulin, which we continued). In addition to protocol differences it may also be important to consider that use of different cell lines and different PSC culture conditions (Muguruma et al. used feeder-dependent culture, while we used feeder-serum free culture) could have played a role. More experiments would be necessary to determine which were the most important factors leading to the differences in our results.

### Useful for quick minimal factor 3D modeling

The protocol reported here offers a quick and easy method for 3D culture, with results comparable to more expensive or complex techniques. It holds potential for research into development of the early cerebellar neuroepithelium, or as a base to generate more mature cortical structures. Further, as our results suggest intrinsic factors were sufficient for regional patterning, these procedures may support optimization of minimal factor protocols for other brain regions.

## MATERIALS AND METHODS

### Prepared medium

Neural maintenance medium (NMM) (for 1 liter):

DMEM/F12, Glutamax (500 ml, Life Technologies), Neurobasal medium (500 ml, Life Technologies), N2 supplement (1×, Life Technologies), B27 supplement (1×, Life Technologies), insulin (5 μg ml^−1^, Imgen), L-glutamine (1.5 mM, Life Technologies), non-essential amino acids (NEAA, 100 µM, Life Technologies), penicillin/streptomycin (100 U l^−1^, Life Technologies), beta-mercaptoethanol (10 µM, Life Technologies). Medium was stored at 4°C and used within 3 weeks.

### Human PSC lines: generation, culture and passaging

Human ESCs were obtained from WiCELL (line H01). Three human iPSC control lines (hvs51, 60 and 88) were generated by reprogramming fibroblasts from three healthy human patients (fibroblasts were derived from anonymous, non-identifiable donors and therefore exempt from Institutional Review Board approval), using a lentivirus containing traditional reprogramming factors OCT4, SOX2, KLF4 and C-Myc (Warlich et al., 2011). Human iPSC clones were characterized for pluripotency by alkaline phosphatase, by ICC for OCT3/4, TRA-1-60, TRA-1-81 and SSEA4, and by RT-PCR analysis for TDGF1, UTF1, DNMT3B, REX1, SALL4, DPPA4, DPPA2, ESG1 and reference gene EIF4G2 (Fig. S3). To test differentiation of human iPSCs, the embryoid body formation assay was performed. The iPSC derivatives were tested for expression of germ layer markers: CD31 (mesoderm), MAP2 (ectoderm) and SOX17 (endoderm). The karyotype of all human iPSC clones was normal.

Human PSCs were maintained in feeder free culture using E8 medium on GelTrex (GT, LifeTechnologies) coated six-well plates (Greiner) at 37°C (5% CO_2_). Colonies were passaged every 3-4 days using standard EDTA passaging protocol. Briefly, each well was washed twice with 1 ml of 0.5 mM EDTA, followed by incubation in 1 ml of 0.5 mM EDTA for 2-3 min at 37°C (5% CO_2_). EDTA was aspirated and cells resuspended in E8 medium+ROCK Inhibitor (RI, 10 μM, SelleckChem) using a 5 ml pipette before replating (1:1 to 1:12, depending on confluency).

To remove build-up of differentiated cells in PSC culture, or for start of differentiations (where smaller colony fragments were desired), PSCs were passaged using a gentle EDTA passaging method (G-method). In this case, each well was washed twice with 1 ml 0.5 mM EDTA, followed by incubation in 1 ml PBS for 4-9 min at 37°C (5% CO_2_). Cells were released by tapping gently on the plate and transferred using a 5 ml pipette to a 15 ml centrifuge tube for gravity separation (∼10 min at room temperature). PBS was aspirated and cells resuspended in appropriate medium+RI (10 μM) before plating.

All PSC lines were monitored for signs of infection, and routinely screened for mycoplasma every six months.

### 3D cerebellar differentiation protocol

On day 0, 2-3 wells (depending on confluency) of human PSCs were passaged using the G-method, with cells resuspended in Neural Maintenance Medium (NMM)+FGF2 (4 ng ml^−1^, Peprotech)+RI (10 µM), and distributed to anti-adhesive (Poly-HEMA, Sigma, P3932-10G) coated 6WPs in a 2 or 3:1 ratio (with 2.5 ml medium per well). Cells were left alone for three days, without addition or change of medium, to allow for formation of embryoid bodies (EBs). A starting population of >500 EBs is important for successful completion of the differentiation. On day 3, culture medium was switched to NMM+FGF2 (4 ng ml^−1^) and maintained for four days to continue growth of EBs. On day 7, culture medium was switched to NMM+RA (1 µM, Sigma)+FGF8B (100 ng ml^−1^, Peprotech) +FGF2 (4 ng ml^−1^) and maintained for seven days to promote neural cell identity and initiate patterning for cerebellar fates. On day 14, culture medium was switched to NMM+FGF8B (100 ng ml^−1^) +FGF4 (100 ng ml^−1^, R&D Systems) +FGF2 (20 ng ml^−1^) and maintained for three days to reinforce growth and patterning for cerebellar cell identity. On day 17, culture medium was switched to NMM+FGF8B (100 ng ml^−1^) and maintained for five days to continue reinforcing cerebellar cell identity. On day 21, culture medium was switched to NMM+Brain Derived Neurotrophic Factor (BDNF, 100 ng ml^−1^, Peprotech) +Glial Derived Neurotrophic Factor (GDNF, 10 ng ml^−1^, Peprotech) and maintained for seven days to continue culture without patterning factors. On day 28, culture medium was changed to NMM+BDNF (100 ng ml^−1^) +GDNF (10 ng ml^−1^) +Smoothened Agonist (SAG, 3 ng ml^−1^, Cayman)+Neurotrophic Factor 3 (NT3, 100 ng ml^−1^, Peprotech) +KCl (25 mM, Sigma) and maintained for seven days to mature and assist the survival of cerebellar cells, particularly granule cells.

Throughout differentiation, medium was kept at 2.5 ml per well (of 6WP) and cells stored at 37°C (5% CO_2_). After the first three days, medium was refreshed every two days. Refreshing and switching medium was performed by removing 2 ml of old medium and adding 2 ml of fresh (appropriate) medium. Accumulation of dead cells or strong color change in medium (to yellow) could indicate a need to refresh medium early (including during the first three days). Dead cells were removed from cultures by gravity sorting. Briefly, cells were transferred with 5 ml pipette to a 15 ml centrifuge tube and allowed to sit for 10 min at room temperature, supernatant aspirated, and cells resuspended in appropriate medium for transfer to a new plate.

At day 35, all products from one differentiation were collected, fixed and frozen per cell line for analysis by immunofluorescence imaging. As such every experiment included a number of aggregates that were analyzed. The differentiations were repeated using four independent PSC lines: ESC line H01 (*n*=5), iPSC line hvs51 (*n*=1), iPSC line hvs60 (*n*=3) and iPSC line hvs88 (*n*=4). The *n*-value represents the number of experiments (differentiations) run on that specific cell line.

### 3D growth factor testing protocol

To test the importance of different growth factors on differentiation, human ESCs were run through the same general 3D differentiation protocol described above, but with four altered medium conditions. Condition A contained no growth factors (all medium content except FGF2, FGF4 and FGF8). Condition B contained only FGF4 and FGF8. Condition C contained only FGF2. Condition D contained FGF2, FGF4 and FGF8 (identical to the original 3D cerebellar protocol; Fig. 1A).

At day 35, all products of one differentiation were collected, fixed and frozen per condition for analysis by immunofluorescence imaging. The experiment was repeated for all conditions (A-D) a total of three times (*n*=3) using human ESC line H01. The *n*-value represents the number of experiments (differentiations).

### 3D differentiation product fixation and immunocytochemistry

Products of one differentiation were collected together, washed with PBS (3×) and fixed in 4% paraformaldehyde (PFA, Electron Microscopy Science) overnight at 4°C. The next day products were washed with PBS (3×) and allowed to sink in 30% sucrose solution overnight at 4°C. Finally, collected products were quick-frozen using OCT (Sakura Finetek Europe BV) and cryosectioned at 11 μm (Leica cryostat). For every marker we analyzed 2-3 serial sections containing 1-10 (single or merged) aggregate products per section.

ICC was performed and immunostained sections visualized as previously described (Dooves et al., 2016), using primary antibodies targeting DCX (1:1000; Cell Signaling; #4604), NeuN (1:500; Millipore; MAB377), PAX6 (1:50; Hybridoma Bank; PAX6-s), TBR2 (1:500; Abcam; AB23345), KIRREL2 (1:200; ProSci; #7971), and ZIC1 (1:200; Sigma; HPA004098) and Alexa Fluor 488- or 594-tagged secondary antibodies (1:1000; Life Technologies; A11001, A11005, A11008, A11012), with DAPI (1:1000; Sigma; D3571) counterstaining. Note: TAG1 (Hybridoma Bank; 4D7/TAG1-s) and ATH1 (donation from Prof. Muguruma) antibodies were also used for ICC but failed in our hands and so not mentioned in Results and Discussion.
